# Biodiversity survey and estimation for line-transect sampling

**DOI:** 10.3389/fpls.2023.1159090

**Published:** 2023-11-10

**Authors:** Youhua Chen, Ren-Hong Wang, Tsung-Jen Shen

**Affiliations:** ^1^ China-Croatia “Belt and Road” Joint Laboratory on Biodiversity and Ecosystem Services, Chengdu Institute of Biology, Chinese Academy of Sciences, Chengdu, China; ^2^ Graduate Institute of Statistics & Department of Applied Mathematics, National Chung Hsing University, Taichung, Taiwan

**Keywords:** biodiversity survey, limited sampling efforts, non-independence, Markov chain, line transects

## Abstract

Conducting biodiversity surveys using a fully randomised design can be difficult due to budgetary constraints (*e.g.*, the cost of labour), site accessibility, and other constraints. To this end, ecologists usually select representative line transects or quadrats from a studied area to collect individuals of a given species and use this information to estimate the levels of biodiversity over an entire region. However, commonly used biodiversity estimators such as Rao’s quadratic diversity index (and especially the Gini–Simpson index) were developed based on the assumption of independent sampling of individuals. Therefore, their performance can be compromised or even misleading when applied to species abundance datasets that are collected from non-independent sampling. In this study, we utilise a Markov chain model and derive an associated parameter estimator to account for non-independence in sequential sampling. Empirical tests on two forest plots in tropical (Barro Colorado, Island of Panama) and subtropical (Heishiding Nature Reserve of Guangdong, China) regions and the continental-scale spatial distribution of *Acacia* species in Australia showed that our estimators performed reasonably well. The estimated parameter measuring the degree of non-independence of subsequent sampling showed that a non-independent effect is very likely to occur when using line transects to sample organisms in subtropical regions at both local and regional spatial scales. In summary, based on a first-order Markov sampling model and using Rao’s quadratic diversity index as an example, our study provides an improvement in diversity estimation while simultaneously accounting for the non-independence of sampling in field biodiversity surveys. Our study presents one possible solution for addressing the non-independent sampling of individuals in biodiversity surveys.

## Introduction

For various reasons (*e.g.*, a limited research budget and field-site inaccessibility), biodiversity surveys are usually conducted according to the most convenient (haphazard) method. Such a common practice can have a significant impact on post-survey data analyses because most of the commonly used ecological methods and biodiversity indices implicitly assume that the data are collected independently. For example, in the estimation of species richness using individual-based data sets, a common assumption is that individuals of different species are sampled independently and randomly (thus following a multinomial distribution) (Zahl, 1977; Chao and Lee, 1992; Shen et al., 2003; Shen et al., 2017).

However, this assumption can be easily violated in practice (Hurlbert, 1984; Heffner et al., 1996; Chen et al., 2019; Chen and Shen, 2020; Chen et al., 2021) because individuals collected from selected sites within a study region are in many cases not independent. This is particularly true when we sample species near rivers, roads, and the edges of forests (Chen, 2014; Chen et al., 2015) to reduce the workload. Moreover, some natural phenomena may also cause biodiversity sampling to be spatially dependent; for example, when collecting seeds from sink habitats, some species from the source may have arrived more easily due to greater dispersal ability or environmental suitability (Chen et al., 2018a).

Some biodiversity indices and statistical methods, including the estimation of species richness in which independent sampling is commonly assumed, run the risk of inaccurately estimating species diversity; the estimation power of these methods can then be field context dependent. To this end, it is necessary to develop statistical methods that can consider non-independent sampling of individuals (Chen et al., 2019; Song et al., 2020). In this study, we utilise a Markov model (Solow, 2000; Chen et al., 2019) that can effectively incorporate positive correlations of individual sampling in a multi-species setting and derive relevant statistical quantities to provide parameter estimation for the model.

To develop the Markov model and derive relevant accurate estimators for addressing the non-independent sampling issue when using line transects for biodiversity surveys, we employ Rao’s quadratic diversity index (Rao, 1982; Nayak, 1986; Botta-Dukat, 2005; Ricotta, 2005a; Rao, 2010) as a demonstration. Rao’s quadratic diversity index was developed based on a totally independent sampling of individuals and has been widely applied in community ecology, from functional to phylogenetic studies (Botta-Dukat, 2005; Ricotta, 2005a; Ricotta, 2005b; Mouchet et al., 2010; Chen et al., 2018b). Rao’s index can be easily reduced to another well-known biodiversity index: the Gini–Simpson index (Simpson, 1949; Magurran, 2004; Jost, 2006; Chen, 2015; Chen et al., 2018b). Moreover, it can be shown to be identical to Nei’s genetic diversity index (Nei, 1973; Nei and Li, 1979; Nei, 1987; Nei and Kumar, 2000), thus making the index broadly applicable in the estimation of biodiversity and various other settings.

In summary, the present study aims to demonstrate that non-independence can emerge, particularly when the sample size is small and a sequential sampling protocol is applied. Accordingly, we hypothesise that when non-independence of sampling exists, common biodiversity indices may be biased when routine calculation formulae are applied. In this context, we introduce a first-order Markov transition model to characterise the sequential feature of line-transect sampling and adjust the biased biodiversity indices. We use a widely used index, Rao’s quadratic diversity index, as a case study, even though its estimation bias is generally low. Rao’s quadratic diversity index has broad application in ecological studies, and it can be shown to be identical to Nei’s nucleotide diversity index. Therefore, our proposed Markov model-based adjustment of biodiversity indices may have application potential in molecular ecology studies. Finally, and most importantly, we introduce our models in terms of distance; however, the application of the sampling scheme can be broadened, *e.g.*, in terms of time.

## Materials and methods

### A Markov model for non-independent sequential sampling of organisms

Suppose that the true relative abundances of *S* species in a community are given by $p_{i}$ , i=1,…,S with $\sum_{i=1}^{S}p_{i}=1$ . Moreover, suppose that an ecologist will consecutively sample *m* individuals one by one from the community, in which the sampling sequence is given by $Z_{k},\;k=1,2,\ldots ,m$ (representing the species label of the *k*th sampled individual). Specifically, the underlying probability model of the sampling process is that the first individual is assumed to be sampled randomly based on each species’ relative abundance (Solow, 2000; Chen et al., 2019), *i.e.*,


(1) $$P\left(Z_{1}=i\right)=p_{i},\;\;$$

and the subsequently sampled individuals follow the transition probabilities of a first-order Markov chain (Solow, 2000; Chen et al., 2019):


(2) $$P\left(Z_{k}=j|Z_{k-1}=i\right)=\{\begin{array}{c} \left(1-\pi \right)p_{i}+\pi ,\;\;\;j=i\\ \left(1-\pi \right)p_{j},\;\;j\neq i \end{array}\;.$$

These probabilities are elements derived from an S×S Markov transition matrix. The probability for j=i in Eq. 2 represents the diagonal elements of the matrix. Note that the parameter π in Eq. 2 has a value ranging from 0 to 1, describing the non-independent sampling of two subsequent individuals from different or the same species. If π=0 , the sampling procedure is independent. By contrast, if π=1 , the sampling procedure will only result in individuals from a single species in the community.

The number of individuals of species *i* observed in the sample can be estimated as


(3) $$N_{i}=\sum_{k=1}^{m}I(Z_{k}=i)\;,$$

where $I\left(Z_{k}=i\right)=1$ if the *k*th selected individual belongs to species *i*, and $I\left(Z_{k}=i\right)=0$ , otherwise. Note that for a given species *i*, $I\left(Z_{k}=i\right)$ , k=1,2,…,m are not independent. Additionally, for any two distinct species *i* and *j* along with a large *m*, the covariance of their abundances can be estimated as


(4) $$Cov\left(N_{i},N_{j}\right)\approx -\delta mp_{i}p_{j}\;,\;$$

where $\delta =\frac{1+\pi }{1-\pi }$ . A detailed derivation of Eq. 4 is provided in the Supplementary Material.

### Rao’s quadratic diversity index

As mentioned above, Rao’s quadratic diversity index is one of the most widely applied indices in studies of phylogenetic and functional community ecology (Botta-Dukat, 2005; Chen, 2015; Chen et al., 2018b). Here, the calculation involves summing the species’ pairwise distances (*e.g.*, phylogenetic distance) weighted by the product of both species’ relative abundances. The formula is given by (Botta-Dukat, 2005; Ricotta, 2005b; Gusmao et al., 2016; Chen et al., 2018b)


(5) $$Q\left(p\right)=\sum_{i\neq j}d_{ij}p_{i}p_{j},\;$$

where $d_{ij}$ is the species’ pairwise distance. Here, the pairwise distance can be the phylogenetic distance from a time-calibrated tree when estimating phylogenetic diversity or genetic distance when measuring genetic diversity. Furthermore, $Q\left(\hat{p}\right)$ is identical to Nei’s genetic diversity index (see **Supplementary Material**).

When $d_{ij}=1$ , for i≠j ; otherwise, $d_{ij}=0$ , Rao’s quadratic diversity index simply becomes the Gini–Simpson index denoted by $\Delta =1-\sum_{i=1}^{S}p_{i}{\;}^{2}$ (Simpson, 1949; Magurran, 2004; Jost, 2006), another well-known diversity index (Pielou, 1969; Pielou, 1977; Krebs, 1989; Magurran, 2004; Chen, 2015). For a local assemblage with *m* individuals *independently* sampled from a community, the observed relative abundance vector $\hat{p}=\left({\hat{p}}_{1},\ldots ,{\hat{p}}_{S}\right)$ (where ${\hat{p}}_{i}=N_{i}/N$ and $N_{i}$ is the observed abundance of species *i* in the local assemblage) is usually used for estimating the index and is denoted as $Q\left(\hat{p}\right)$ . The unbiased index is computed as $Q^{U}\left(p\right)=\sum_{i\neq j}d_{ij}\frac{N_{i}\left(N_{i}-1\right)}{m\left(m-1\right)}$ . Accordingly, the two well-known estimators for the Gini–Simpson index are the maximum likelihood (ML) estimator Δ^=1−∑i=1S(Nim)2 and the unbiased estimator ${\hat{\Delta }}_{U}=1-\sum_{i=1}^{S}\frac{N_{i}\left(N_{i}-1\right)}{m\left(m-1\right)}$ (Chen et al., 2018b).

In our study, we calculated Rao’s quadratic diversity index (and the Gini–Simpson index in particular) to demonstrate how non-independent sampling may bias the estimate using a line-transect sampling strategy and how the estimate can be improved using a Markov model.

### Parameter estimation

Solow (2000) provided an effective and rapid estimator for the non-independence parameter π . Specifically, the parameter measures the probability of observing two subsequently sampled individuals of the same species, *i.e.*, the estimator of π can be mathematically expressed as follows:


(6) $$v=\frac{1}{m-1}\sum_{k=2}^{m}\sum_{i=1}^{S}I\left(Z_{k}=i,Z_{k-1}=i\right).$$

Note that the denominator m−1 in Eq. 6 is the total number of adjacent pairs in a sample of m individuals. The expectation of Eq. 6 can be expressed as:


(7) $$E\left(v\right)=\left(1-\text{π}\right)\sum_{i=1}^{S}p_{i}^{2}+\text{π}.\;$$

Therefore, Solow’s (2000) estimator v is expected to overestimate π , and the magnitude of the bias is the first term of the right-hand side of Eq. 7. We will derive a nearly unbiased estimator for π , in which v is still useful and valid for some scenarios.

From Eqs. 4 and 5 with $d_{ij}=1$ , for i≠j ; otherwise, $d_{ij}=0$ , an alternative expression of the Gini–Simpson index is


(8) $$\hat{\text{Δ}}\approx \frac{m^{2}-\sum_{i=1}^{S}E\left(N_{i}^{2}\right)}{m\left(m-\text{δ}\right)},$$

from which, in combination with an estimator for π (or equivalent to δ ) introduced later, we propose a nearly unbiased estimator of $\hat{\text{Δ}}$ by estimating $E\left(N_{i}^{2}\right)$ as $N_{i}^{2}$ . For deriving an estimator of π, two equations (based on the method of moments) can be constructed from Eqs. 7 and 8 by removing the expectation operators. After some algebraic manipulation of the two equations, the explicit unbiased estimator of π is:


(9) $$\hat{\pi }=1-\frac{m\left(m+1\right)\left(1-v\right)+\sqrt{m^{2}{\left(m+1\right)}^{2}{\left(1-v\right)}^{2}-8m^{3}\left(1-v\right)\hat{\text{Δ }}}}{2m^{2}\hat{\text{Δ}}},\;$$

provided the term inside the root sign is non-negative, and the resulting value is not larger than the upper bound v; , otherwise, for simplicity, we suggest using v instead. Note that $\hat{\text{Δ}}$ in Eq. 9 is $\hat{\text{Δ}}=1-\sum_{i=1}^{S}{\left(\frac{N_{i}}{m}\right)}^{2}$ . Accordingly, based on Eq. 9, the associated estimator for δ is $\hat{\text{δ}}=\frac{1+\hat{\text{π}}}{1-\hat{\text{π}}}$ .

As to the estimation of Rao’s quadratic diversity index, using Eq. 4, after some algebra we derive a nearly unbiased estimator of Q(p) , under the assumption of non-independent sampling as follows:


(10) $${\hat{Q}}^{M}\left(p\right)=\sum_{i\neq j}\frac{d_{ij}N_{i}N_{j}}{m\left(m-\hat{\text{δ }}\right)}\text{ .}$$

The derivation of Eq. 10 can be found in the Supplementary Material. Notably, this estimator covers the typical unbiased estimator ${\hat{\text{Δ}}}_{U}$ derived from the random sampling context (Pielou, 1975; Nayak, 1986; Chen et al., 2018b). Specifically, when π=0 (*i.e.*, sampling of individuals is totally independent) or equivalently $\hat{\text{δ}}=1$ , Eq. 10 is the same as the unbiased index.

### Semi-numerical simulation and evaluation

In this study, we used two empirical datasets to perform semi-numerical simulation and evaluation of the performance of different diversity estimators under distinct sampling assumptions. The first dataset comprised biomass data of plant communities sampled from ultramafic soils of Tuscany, central Italy (Chiarucci et al., 1998; Ricotta, 2005a; Chen et al., 2018b). In this dataset, because only a taxonomic classification tree for 26 plant species was available, we assigned an equal weight (20) to each classification linkage connecting a higher taxonomic unit (*e.g.*, family) to a subsequent lower taxonomic unit (*e.g.*, genus) (Ricotta, 2005a). The pairwise species distance $d_{ij}$ simply sums all of these equal weights from the most common taxonomic unit to each pair of species (Chen et al., 2018b). To make the calculation of Rao’s index applicable in the semi-numerical tests, we simply assumed that a species’ relative abundance was proportional to the total biomass recorded for that species (Chen et al., 2018b).

The second dataset was derived from the abundance and distribution of the Phyllostomid (leaf-nosed bats) from Selva Lacandona habitats in Chiapas, Mexico (Medellin et al., 2000; Allen et al., 2009). The associated phylogenetic tree for 34 genera of Phyllostomidae (Baker et al., 2003; Allen et al., 2009) was used to compute phylogenetic distances between pairs of genera. Again, the relative abundance of each genus in this dataset was used for the semi-numerical simulations.

For analyzing the impact of non-independent sampling on the assessment of biodiversity, the true value of the non-independence parameter π for the two data sets was set to 0.1, 0.25, 0.4, 0.55, and 0.7. In addition, we used four sample sizes (*m* = 50, 75, 100, and 125) in the simulation study to reflect their effect on the estimation of parameters associated with calculating the biodiversity indexes.

### Empirical tests

We used the stand mapping data from a Barro Colorado Island (BCI) tropical forest plot to investigate the potential influence of non-independent sampling when travelling across line transects to conduct a biodiversity assessment for the entire forest plot. The BCI forest plot has an area of 50 ha ( 1,000×500 m) and was established by Stephen Hubbell and Robin Forster in 1980 (Condit et al., 1996; Condit, 1998; Hubbell et al., 1999; Condit et al., 2002; Condit et al., 2012). In the present study, we used the 2005 census data. Only individual trees or shrubs with a diameter at breast height larger than 10 mm were included (Chen et al., 2018a; Chen et al., 2019; Chen et al., 2021). In addition to the BCI plot, we also utilised the Heishiding (HSD; 50 ha with 1,000 × 500 m; 2011 census) plot located within the Heishiding Provincial Reserve in the Guangdong province of China (Yin and He, 2014), which represented a subtropical forest community. Finally, for testing the potential non-independent sampling issue at a very broad-spatial scale, we also utilised the distribution of 508 *Acacia* species in Australia, an ideal region for studying and assessing large-scale biodiversity and biogeographic patterns due to the nearly complete herbarium records and collection (Mishler et al., 2014; Bloomfield et al., 2018). Of the 1000 *Acacia* species described in Australia, only a very small fraction occur outside the territory (Mishler et al., 2014).

**Figure 1:**
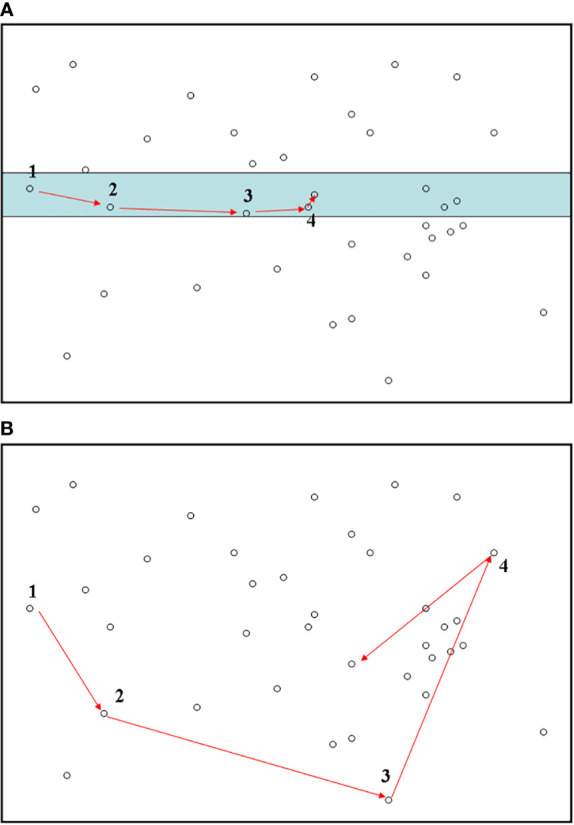
Biodiversity sampling using a line-transect method **(A)** in the present study. The fully random sampling method **(B)** is also provided for comparison. For each subplot, numbers along with arrows stand for the orders of individuals when conducting the sampling. The shaded area in subplot **(A)** represents the line-transect band. In subplot **(B)**, to conduct totally random sampling of individuals, it would be necessary to perform a random walk across the entire region. This sampling strategy would be labour-expensive and time-consuming.

We implemented the line transect method as a cost-effective strategy to sample species individuals that may present an apparent spatially dependent sampling structure (**Figure 1**). In detail, a line transect with a chosen small width (1 m) was randomly placed on the two forest plots (HSD and BCI) to sample tree individuals and on the territory of Australia (the width of a specific line transect was now larger, usually 2 km) to sample the individuals of *Acacia* species. The length of the line transect was as yet undetermined and was subsequently determined based on the pre-designed sample size as described below.

To set a reference point, the starting point of the line transect was randomly located in an arbitrary direction. However, for convenience, the starting point of the sampling was usually selected on the edge of the study region (*e.g.*, **Figure 1A**). All individuals within the line transect band were surveyed sequentially according to the distance of each individual from the reference point (**Figure 1**). Specifically, among all individuals in the line transect band, the initial organism was the one having the shortest distance from the reference point; we then searched for the second individual (regardless of species identity) that had the minimal distance from the reference point other than the first, and so forth. Note that previously measured individuals were not sampled repeatedly. If there were multiple neighboring individuals with the same minimal distance, we randomly chose one. This way of identifying subsequent individuals of species along a line transect is very economical and labour-inexpensive, as our sampling strategy is equivalent to finding nearest neighbors in sequence along a line transect. The sampling is complete when the required sample size is fulfilled; that is, we only record the first *m* individuals along the line transect. As a comparison, the fully random sampling method—in which all individuals have to be randomly chosen across the entire target area (irrespective of how far away the two successive individuals are)—is expensive and labour-intensive and therefore often prohibitive for ecologists to implement (**Figure 1B**).

When applying the line-transect sampling to each of the three empirical datasets, in considering the potential confounding influence of sample size, we employed seven sample sizes as *m* = 50, 100, 500, 1,000, 2,000, 5,000, and 8,000. For each sample size, 5,000 replicates (other numbers of replicates >500 would be sufficient) of line-transect sampling were conducted. For each replicate, if the number of individuals sampled from a single line transect (across the boundary of the sampling region, *e.g*., the territory of Australia) did not reach the required sample size, using the endpoint of the previous line transect as the starting point, we placed a new line transect onto the target area to continue subsequent sampling of individuals (**Figure 1A**). This step was repeated until the required sample size was reached (**Figure 1A**).

To quantify estimation accuracy (Chen et al., 2018b) and compare the performance of different estimators for each estimator considered in this study, we computed the average (Avg), statistical bias (BIAS), and root mean squared error (RMSE) using the resulting 5,000 replicate estimates. It should be noted that the variance of the point estimate (also the reciprocal of precision) can be measured by the difference between the squared RMSE and the squared bias.

## Results

The two semi-numerical studies demonstrated that the estimation of the non-independence parameter π was very accurate (**Tables 1**, **S1**). Therefore, it would be reasonable and reliable to apply the estimated parameters of the Markov model to evaluate the impacts of different spatial sampling methods in practice.

**Table 1:** Estimate averages, averaged bias (BIAS), and root mean squared error (RMSE) of the original Solow’s estimator and the proposed estimators for the non-independence parameter π in the semi-numerical test using abundance information for Phyllostomid (leaf-nosed bats) from Selva Lacandona habitats in Chiapas, Mexico.

*m*	π	Solow’s estimator: *v*			Proposed estimator: π		
		Avg	BIAS	RMSE	Avg	BIAS	RMSE
50	0.10	0.129	0.029	0.056	0.100	0.000	0.050
75		0.129	0.029	0.048	0.100	0.000	0.040
100		0.128	0.028	0.043	0.099	-0.001	0.034
125		0.129	0.029	0.042	0.100	0.000	0.031
50	0.25	0.274	0.024	0.069	0.251	0.001	0.067
75		0.274	0.024	0.057	0.250	0.000	0.053
100		0.274	0.024	0.050	0.250	0.000	0.046
125		0.273	0.023	0.047	0.249	-0.001	0.042
50	0.40	0.418	0.018	0.073	0.400	0.000	0.074
75		0.418	0.018	0.060	0.400	-0.000	0.060
100		0.419	0.019	0.053	0.400	0.000	0.051
125		0.419	0.019	0.049	0.400	-0.000	0.046
50	0.55	0.564	0.014	0.071	0.552	0.002	0.073
75		0.564	0.014	0.059	0.550	0.000	0.060
100		0.564	0.014	0.051	0.551	0.001	0.051
125		0.563	0.013	0.047	0.549	-0.001	0.046
50	0.70	0.710	0.010	0.065	0.703	0.003	0.067
75		0.711	0.011	0.053	0.703	0.003	0.054
100		0.710	0.010	0.046	0.702	0.002	0.046
125		0.709	0.009	0.042	0.700	0.000	0.043

Regarding the application of Rao’s quadratic diversity index, there were basically no differences between the estimated and true values when the non-independent Markov model was used, particularly when the sample size was large (**Tables 2**, **S2**). For comparison, if there was strong evidence of a non-independent pattern of sequentially sampled individuals (*i.e.*, *π* = 0.75), the bias induced by both biased and unbiased Rao’s indices derived from totally independent sampling of individuals was much higher than the proposed estimators derived from the assumption of non-independent sampling (**Tables 2**, **S2**). The comparative studies for the Gini–Simpson index had similar results: the proposed index based on non-independent sampling had the best performance (**Tables S3**, **S4**).

**Table 2:** Estimate average, average bias (BIAS), and root mean squared error (RMSE) of the original unbiased Rao’s index and the proposed estimators from an ecological community (*i.e.*, 34 Phyllostomid bat genera abundances in Selva Lacandona habitats).

*m*	*π*	MLE: $Q\left(\hat{p}\right)$			Unbiased: ${\hat{Q}}^{U}\left(p\right)$			Proposed: ${\hat{Q}}^{M}\left(p\right)$		
		Avg	BIAS	RMSE	Avg	BIAS	RMSE	Avg	BIAS	RMSE
Bat data in Selva lacandona habitats: *Q*(*p*)=177.3										
50	0.10	173.0	-4.4	5.3	176.5	-0.8	3.2	177.3	-0.0	3.1
75		174.4	-2.9	3.8	176.8	-0.5	2.5	177.3	0.0	2.5
100		175.2	-2.2	3.0	176.9	-0.4	2.2	177.3	-0.0	2.1
125		175.6	-1.7	2.5	177.0	-0.3	1.9	177.3	-0.0	1.9
50	0.25	171.5	-5.8	6.9	175.0	-2.3	4.4	177.5	0.2	3.8
75		173.4	-3.9	4.9	175.8	-1.0	3.4	177.4	0.1	3.0
100		174.4	-2.9	3.9	176.2	-1.2	2.8	177.4	0.0	2.6
125		175.0	-2.3	3.2	176.4	-0.9	2.4	177.4	0.1	2.2
50	0.40	169.3	-8.0	9.3	172.8	-4.6	6.6	177.8	0.5	4.8
75		171.9	-5.5	6.6	174.2	-3.2	4.9	177.5	0.1	3.7
100		173.2	-4.1	5.1	175.0	-2.4	3.8	177.4	0.1	3.0
125		174.0	-3.3	4.2	175.4	-1.9	3.3	177.4	0.0	2.7
50	0.55	165.5	-11.8	13.4	168.9	-8.5	10.6	178.3	1.0	6.5
75		169.4	-79	9.2	171.7	-5.6	7.3	177.8	0.4	4.7
100		171.4	-6.0	7.1	173.1	-4.3	5.7	177.6	0.3	3.9
125		172.5	-4.9	5.9	173.9	-3.5	4.8	177.4	0.1	3.4
50	0.70	158.4	-18.9	21.0	161.7	-15.7	18.3	180.7	3.3	10.9
75		164.3	-13.0	14.6	166.5	-10.8	12.8	178.5	1.2	7.1
100		167.6	-97	11.1	169.3	-8.0	9.7	178.1	0.7	5.0
125		169.5	-7.8	9.0	170.9	-6.4	7.9	177.8	0.5	4.6

In the three empirical datasets with varying spatial sampling scales, the non-independence parameter was estimated to be low (approximately 0.047) when conducting line-transect sampling in the BCI plot (**Table S5**), while being relatively high when conducting line-transect sampling in both the HSD forest plot (approximately 0.269; **Table S6**) and for Australian *Acacia* species (approximately 0.327; **Table S7**). Moreover, the estimation of the non-independence parameter π was not influenced by the largest sample sizes in all three empirical datasets (**Tables S1**, **S5–S7**). When the sample size was sufficiently large (*e.g.*, *m* = 5,000 or 8,000), the estimated π values became asymptotically stable, regardless of which dataset was tested (**Tables S5**–**S7**).

**Figure 2:**
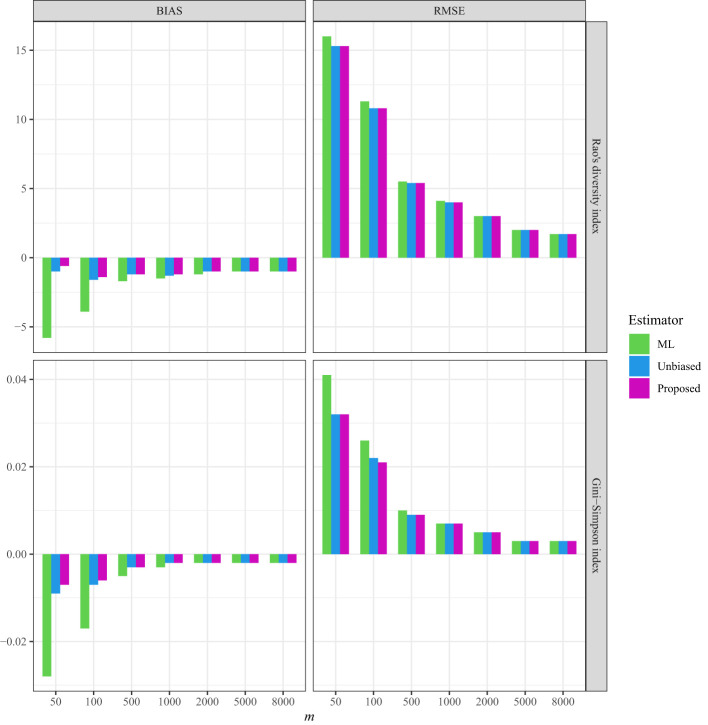
The average bias (BIAS) and root mean squared error (RMSE) of the ML (maximum likelihood) estimator, the unbiased Rao’s index, and the proposed estimators for the tree data sampled from the line transects in the 50-ha BCI forest plot.

**Figure 3:**
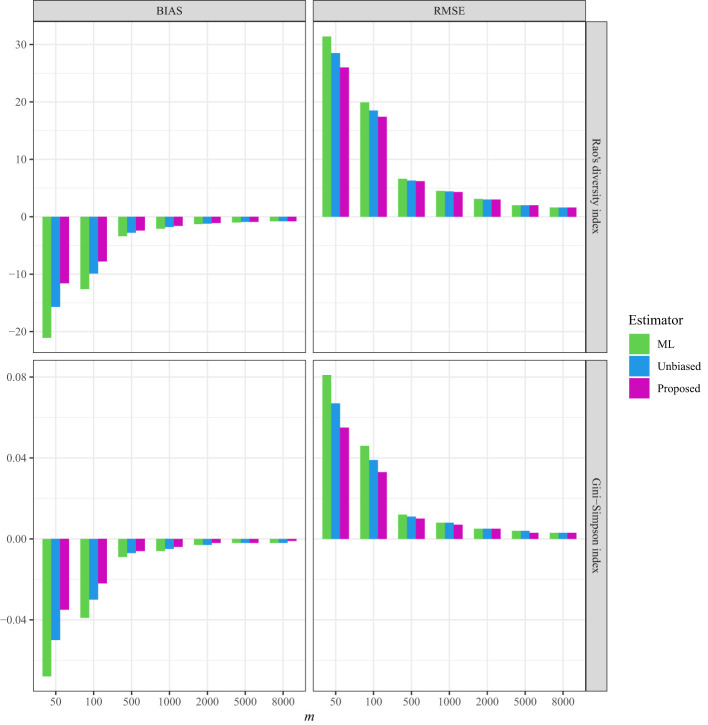
The average bias (BIAS) and root mean squared error (RMSE) of the ML (maximum likelihood) estimator, the unbiased Rao’s index, and the proposed estimators for the tree data sampled from the line transects in the 50-ha HSD forest plot.

**Figure 4:**
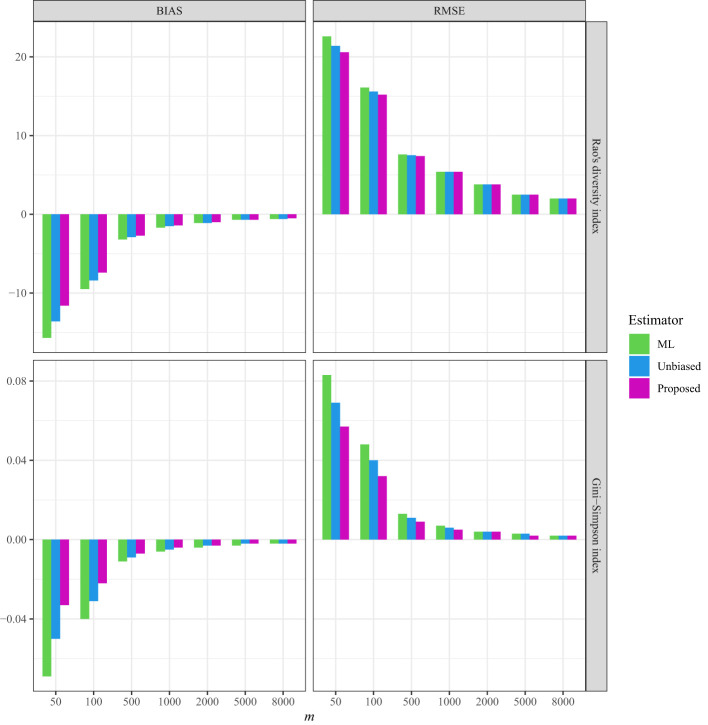
The average bias (BIAS) and root mean squared error (RMSE) of the ML (maximum likelihood) estimator, the unbiased Rao’s index, and the proposed estimator from the line transects in Australia for *Acacia* species.

As a comparison, in all three datasets, the bias and RMSE were quite low when the non-independence parameter was incorporated into the Markov sampling model (**Figures 2**–**4** and **Tables S5**–**S7**). Moreover, as expected, when the required sampling size for the line-transect sampling increased, the bias and RMSE approached zero, as the estimated Rao’s quadratic diversity index and the estimated Gini–Simpson index were close to their true values (**Figures 2**–**4** and **Tables S5**–**S7**).

## Discussion

In the empirical tests, the non-independence parameter was estimated to be low for the BCI plot. However, this low number did not necessarily imply that we need not consider the non-independence sampling in forest ecosystems in that region. The estimates of Rao’s quadratic diversity and Gini–Simpson indices were the most accurate when the Markov sampling-derived estimator was used. Additionally, if the data were collected by an independent sampling scheme and applied to the Markov sampling-derived estimator, the result should be similar to the random sampling-derived estimator (*i.e.*, ${\hat{Q}}^{U}\left(p\right)$ for Rao’s quadratic diversity index) as the estimated value of π should not be too large for random walk data. Therefore, the non-independence of sampling did influence the collection of individuals from the diverse spatially explicit sampling methods. Moreover, all Markov sampling-derived estimators remained valid when applied to randomly sampled individuals.

In practice in the field, totally independent or random sampling of individuals over a studied region can be highly impractical and labour intensive, and travel across the region may be required. Consequently, it is important to recognise that the collection of individuals from selected quadrats in the study region will run the risk of over-representing or under-representing some species if their distributions are highly aggregated. Therefore, it is necessary to recognise that the application of previously developed biodiversity estimators that were built upon the assumption of independent sampling of individuals might not be as powerful as assumed and should be used with relevant caveats. As a result, the first-order Markov chain model employed in the present study may be effective in alleviating the compounding effect of limited spatially dependent sampling for ecologists who wish to accurately estimate the biodiversity level of a region.

The present study represents one possible solution to the non-independent sampling issue in the field setting by deriving pertinent unbiased estimators for the Markov model studied. More importantly, we demonstrated that the non-independence issue was likely to exist when conducting line-transect surveys of ecological communities in the real world (as demonstrated by the HSD tree species and Australian *Acacia* species). Finally, the simulation and empirical tests demonstrated that the proposed estimators provide accurate estimates of important biodiversity indices, such as Rao’s quadratic diversity and the Gini–Simpson index.

The Markov model and the associated estimators were developed for cost-effective sampling in practice. In combination with line-transect sampling, the estimators proposed in this study can save ecologists’ time and energy spent in field sampling without compromising the accuracy of biodiversity estimation. If ecologists have sufficient budget and time available, they may opt for field-sampling methods that are labour-intensive, and individuals of a species may be well sampled in an approximately random manner. By contrast, if ecologists wish to reduce the workload and guarantee work safety by only selecting sampling sites that have potentially strong spatial relationships, non-independent sampling of individuals can be expected. In this case, our estimators should be a good alternative. In addition, as mentioned above, our Markov sampling-derived estimators are robust regardless of whether the data were collected from non-independent or independent sampling. Consequently, the methods proposed in the present study are recommended in practical applications from a cost-effective perspective.

Our present method provides avenues for future research. First, we only estimated the quantity $\sum_{i=1}^{S}p_{i}^{2}$ across all the species in the target area. The estimation of the true relative abundance of each species $p_{i}$ may become possible by effectively estimating those unseen species that are not observed in current samples. This is of particular importance given that their estimates will greatly influence the accuracy of estimated biodiversity levels at the regional scale. Previous studies have developed very robust methods for accurately estimating the relative true abundances of unseen species (Good, 1953; Chiu et al., 2014; Chao et al., 2015; Chao et al., 2017). However, these methods may not be appropriate under the context of Markov non-independent sampling. To this end, we call for the development of suitable methods that can effectively alleviate the confounding impact of unseen species in non-independent sampling. Second, there is a knowledge gap concerning the accurate interpretation of the non-independence parameter under spatially explicit sampling scenarios. For example, to what extent is the non-independence parameter related to the spatial non-randomness? Can a single parameter be applicable to the multi-species situation at the community level? How can we explicitly incorporate spatial information in the modelling (*e.g.*., distance of subsequently sampled individuals) and estimation of biodiversity indices? We believe that many interesting questions are open to be explored by ecologists to provide accurate and reliable biodiversity indices in community ecology and conservation studies.

Naturally, as George Box pointed out, “All models are wrong, some are useful.” All statistical models should be used with caution. Our sequential sampling protocol and the associated improvements in the estimate of some well-known diversity metrics have limitations if the sampling conditions do not follow the theoretical assumptions. For example, if sampling areas have very heterogeneous landscape conditions, the power of our proposed estimators (and other estimators as well) will be affected. In addition, if ecologists conducted original field sampling along dry roads but performed the interpolation in boggy areas without roads.

In conclusion, it is important to recognise and understand ecological mechanisms relevant to estimating bias for biodiversity indices, as this may influence accuracy and may lead to incorrect or even misleading comparisons of biodiversity levels between ecological communities (Chen et al., 2018b). The present study suggests that practical cost-effective spatial sampling methods employed in biodiversity surveys can compromise the power of common biodiversity indices, particularly when developed under the simple assumption of independent sampling. To this end, the present study is one of the first to model the non-independent sampling issue in the collection of biodiversity data to provide a more realistic and accurate estimate of biological diversity derived from field-collected ecological data sets.

## Data availability statement

The BCI forest plot dataset is available for the public from the following URL: https://doi.org/10.15146/R3FH61. The HSD forest plot data are available by sending a request to Dr. Fangliang He (fhe@ualberta.ca). The distribution of *Acacia* species in Australia is available from a previous study (Mishler et al., 2014).

## Author contributions

YC and T-JS conceived the research idea and wrote the first draft of the manuscript. YC and R-HW conducted data analyses. All authors contributed to the article and approved the submitted version.

## References

[B1] Allen B. Kon M. Bar-Yam Y. (2009). A new phylogenetic diversity measure generalizing the Shannon index and its application to phyllostomid bats. Am. Nat. 174, 236–243. doi: 10.1086/600101 19548837

[B2] Baker R. Hoofer S. Porter C. van den Bussche R. (2003). Diversification among New World leaf-nosed bats: an evolutionary hypothesis and classification inferred from digenomic congruence of DNA sequence. Occasional Papers Museum Texas Tech Univ. 230, 1–32. doi: 10.5962/bhl.title.156931

[B3] Bloomfield N. Knerr N. Encinas-Viso F. (2018). A comparison of network and clustering methods to detect biogeographical regions. Ecography 41, 1–10. doi: 10.1111/ecog.02596

[B4] Botta-Dukat Z. (2005). Rao’s quadratic entropy as a measure of functional diversity based on multiple traits. J. Vegetation Sci. 16, 533–540. doi: 10.1111/j.1654-1103.2005.tb02393.x

[B5] Chao A. Chiu C. Colwell R. Magnago L. Chazdon R. Gotelli N. (2017). Deciphering the enigma of undetected species, phylogenetics, and functional diversity based on Good-Turing theory. Ecology 98, 2914–2929. doi: 10.1002/ecy.2000 28869780

[B6] Chao A. Hsieh T. Chazdon R. Colwell R. Gotelli N. (2015). Unveiling the species-rank abundance distribution by generalizing the Good-Turing sample coverage theory. Ecology 96, 1189–1201. doi: 10.1890/14-0550.1 26236834

[B7] Chao A. Lee S. (1992). Estimating the number of classes via sample coverage. J. Am. Stat. Assoc. 87, 210–217. doi: 10.1080/01621459.1992.10475194

[B8] Chen Y. (2014). A comparison on the impacts of short-term micro-environmental and long-term macro-climatic variability on structuring beta diversity of microarthrophod communities. J. Asia-Pacific Entomology 17, 629–632. doi: 10.1016/j.aspen.2014.06.006

[B9] Chen Y. (2015). Biodiversity and biogeographic patterns in Asia-Pacific region I: statistical methods and case studies (UAE: Bentham Science Publishers).

[B10] Chen Y. Amundrud S. L. Srivastava D. S. (2015). Spatial variance in soil microarthropod communities: Niche, neutrality, or stochasticity? Ecoscience 21, 1–14. doi: 10.2980/21-(3-4)-3720

[B11] Chen Y. Shen T. (2020). Unifying conspecific-encounter index v and Moran’s I index. Ecography 43, 1902–1904. doi: 10.1111/ecog.05281

[B12] Chen Y. Shen T. Chung H. Shi S. Jiang J. Condit R. . (2019). Inferring multi-species distributional aggregation level from limited line transected-derived biodiversity data. Methods Ecol. Evol. 10, 1015–1023. doi: 10.1111/2041-210X.13197

[B13] Chen Y. Shen T. Condit R. Hubbell S. (2018a). Community-level species’ correlated distribution can be scale-independent and related to the evenness of abundance. Ecology 99, 2787–2800. doi: 10.1002/ecy.2544 30347110

[B14] Chen Y. Wu Y. Shen T. (2018b). Evaluation of the estimate bias magnitude of the Rao’s quadratic diversity index. PeerJ 6, e5211. doi: 10.7717/peerj.5211 30002990 PMC6037161

[B15] Chen Y. Wu Y. Zhou J. Zhang W. Lin H. Liu X. . (2021). Effectively inferring overall spatial distribution pattern of species in a map when exact coordinate information is missing. Methods Ecol. Evol. 12, 971–984. doi: 10.1111/2041-210X.13590

[B16] Chiarucci A. Maccherini S. Bonini I. De Dominicis V. (1998). Effects of nutrient addition on species diversity and ground cover of “serpentine“. vegetation. Plant Biosyst. 132, 143–150. doi: 10.1080/11263504.1998.10654199

[B17] Chiu C. Wang Y. Walther B. Chao A. (2014). An improved non-parametric lower bound of species richness via a modified Good-Turing frequency formula. Biometrics 70, 671–682. doi: 10.1111/biom.12200 24945937

[B18] Condit R. (1998). Tropical forest census plots (Berlin, Germany, and Georgetown, Texas: Springer-Verlag and R. G. Landes Company).

[B19] Condit R. Hubbell S. Foster R. (1996). Changes in a tropical forest with a shifting climate: results from a 50-ha permanent census plot in Panama. J. Trop. Ecol. 12, 231–256. doi: 10.1017/S0266467400009433

[B20] Condit R. Lao S. Perez R. Dolins S. Foster R. Hubbell S. (2012). Barro colorado forest census plot data 2012 version. Center Trop. For. Sci. Database. doi: 10.5479/data.bci.20130603

[B21] Condit R. Pitman N. Leigh E. G. Chave J. Terborgh J. Foster R. B. . (2002). Beta-diversity in tropical forest trees. Science 295, 666–669. doi: 10.1126/science.1066854 11809969

[B22] Good I. (1953). The population frequencies of species and the estimation of population parameters. Biometrika 40, 237–264. doi: 10.1093/biomet/40.3-4.237

[B23] Gusmao J. Brauko K. Eriksson B. Lana P. (2016). Functional diversity of macrobenthic assemblages decreases in repsonse to sewage discharges. Ecol. Indic. 66, 65–75. doi: 10.1016/j.ecolind.2016.01.003

[B24] Heffner R. Butler M. Reilly C. (1996). Pseudoreplication revisited. Ecology 77, 2558–2562. doi: 10.2307/2265754

[B25] Hubbell S. Foster R. O’Brien S. Harms K. Condit R. Wechsler B. . (1999). Light gap disturbances, recruitment limitation, and tree diversity in a neotropical forest. Science 283, 554–557. doi: 10.1126/science.283.5401.554 9915706

[B26] Hurlbert S. (1984). Pseudoreplication and the design of ecological field experiments. Ecol. Monogr. 54, 187–211. doi: 10.2307/1942661

[B27] Jost L. (2006). Entropy and diversity. Oikos 113, 363–375. doi: 10.1111/j.2006.0030-1299.14714.x

[B28] Krebs C. (1989). Ecological methodology (New York: Harper Collins).

[B29] Magurran A. (2004). Measuring biological diversity (Oxford: Blackwell).

[B30] Medellin R. A. Equihua M. Amin M. A. (2000). Bat diversity and abundance as indicators of disturbance in neotropical rainforests. Conserv. Biol. 14, 1666–1675. doi: 10.1111/j.1523-1739.2000.99068.x 35701935

[B31] Mishler B. D. Knerr N. González-Orozco C. E. Thornhill A. H. Laffan S. W. Miller J. T. (2014). Phylogenetic measures of biodiversity and neo- and paleo-endemism in Australian *Acacia* . Nat. Commun. 5, 4473. doi: 10.1038/ncomms5473 25034856

[B32] Mouchet M. Villeger S. Mason N. Mouillot D. (2010). Functional diversity measures: an overview of their redundancy and their ability to discriminate community assembly rules. Funct. Ecol. 24, 867–976. doi: 10.1111/j.1365-2435.2010.01695.x

[B33] Nayak T. (1986). An analysis of diversity using Rao’s quadratic entropy. Sankhya Ser. B 48, 315–330.

[B34] Nei M. (1973). Analysis of gene diversity in subdivided populations. PNAS 70, 3321–3323. doi: 10.1073/pnas.70.12.3321 4519626 PMC427228

[B35] Nei M. (1987). Molecular evolutionary genetics (New York: Columbia University Press).

[B36] Nei M. Kumar S. (2000). Molecualr evolutoin and phylogenetics (Oxford: Oxford University Press).

[B37] Nei M. Li W. (1979). Mathematical model for studying genetic variation in terms of restriction endonucleases. PNAS 76, 5269–5273. doi: 10.1073/pnas.76.10.5269 291943 PMC413122

[B38] Pielou E. (1969). An introduction to mathematical ecology (New York, USA: John Wiley & Sons, Ltd).

[B39] Pielou E. (1975). Ecological diversity (New York: John Wiley & Sons).

[B40] Pielou E. (1977). Mathematical ecology (New York: Wiley).

[B41] Rao C. (1982). Diversity and dissimilarity coefficients-a unified approach. Theor. Popul. Biol. 21, 24–43. doi: 10.1016/0040-5809(82)90004-1

[B42] Rao C. (2010). Quadratic entropy and analysis of diversity. Sankhya 72-A, 70–80. doi: 10.1007/s13171-010-0016-3

[B43] Ricotta C. (2005a). Additive partitioning of Rao’s quadratic diversity: a hierarchical approach. Ecol. Model. 183, 365–371. doi: 10.1016/j.ecolmodel.2004.08.020

[B44] Ricotta C. (2005b). A note on functional diversity measures. Basic Appl. Ecol. 6, 479–486. doi: 10.1016/j.baae.2005.02.008

[B45] Shen T. Chao A. Lin C. (2003). Predicting the number of new species in further taxonomic sampling. Ecology 84, 798–804. doi: 10.1890/0012-9658(2003)084[0798:PTNONS]2.0.CO;2

[B46] Shen T.-J. Chen Y. Chen Y.-F. (2017). Estimating species pools for a single ecological assemblage. BMC Ecol. 17, 45. doi: 10.1186/s12898-017-0155-7 29273049 PMC5741966

[B47] Simpson E. (1949). The measurement of diversity. Nature 163, 688. doi: 10.1038/163688a0

[B48] Solow A. (2000). The effect of dependence on estimating sample coverage. Environmetrics 11, 245–249. doi: 10.1002/(SICI)1099-095X(200003/04)11:2<245::AID-ENV408>3.0.CO;2-S

[B49] Song C. Peacor S. Osenberg C. Bence J. (2020). An assessment of statistical methods for nonindependent data in ecological meta-analyses. Ecology 101, e03184. doi: 10.1002/ecy.3184 32893349

[B50] Yin D. He F. (2014). A simple method for estimating species abundance from occurrence maps. Methods Ecol. Evol. 5, 336–343. doi: 10.1111/2041-210X.12159

[B51] Zahl S. (1977). Jackknifing an index of diversity. Ecology 58, 907–913. doi: 10.2307/1936227

