# Supplementary material for: Biodiversity survey and estimation for line-transect sampling
Source: Front Plant Sci. 2023 Nov 10;14:1159090. doi: 10.3389/fpls.2023.1159090 (PMC10667475; doi:10.3389/fpls.2023.1159090)

**Supporting Information**

**Biodiversity survey and estimation for line-transect sampling**

**Mathematical foundation of the proposed statistical model**

*Some basic statistical properties*

Some key formulas, which are necessary for deriving the unbiased non-independent strength parameter, are provided below. For any integer $k\geq1$, proofs for these key formulas can be easy, and thus omitted for brevity.

1)

2) $P\left( \left. Z_{k+1}=i \right|Z_{k}=i \right)=1-\left( 1-\pi\right)q_{i}=\left( 1-\pi\right)p_{i}+\pi$, where $q_{i}=1-p_{i}; (S1b)$

Note that Eqs. S1a and S1b are directly from transition probabilities of the Markov model in Eq. 2 of the main text.

3)

4) $P\left( \left. Z_{k+i}\neq i \right|Z_{k}=i \right)=\left( 1-\pi\right)q_{i}. (S1d)$.

Eqs. S1c and S1d can be straightforwardly derived from Eqs. S1a and S1b. Because the sampling of the first individual along a line transect is random and proportional to species’ relative abundances, we have $P\left( Z_{1}=i \right)=p_{i}$. For the second sampled individual, based on the above equalities (Eqs. S1a-S1d), we can derive the probability of the individual belonging to species $i$ as

5)

The result $P\left( Z_{k}=i \right)=p_{i}$ for any integer $k\geq3$ can be derived from mathematical induction.

*Derivation of sampling variance of the proposed model*

When sampling *m* individuals along a transect, the number of individuals being sampled for species *i* is denoted by $N_{i}$, for which we can further express it in terms of species labels of the individuals $\left( Z_{k},k=1,2,\ldots,m \right)$ as follows:

where $I\left( Z_{k}=i \right)=1$ if the *k*th sampled individual is from species *i*; $I\left( Z_{k}=i \right)=0$, otherwise.

Its variance thus can be calculated by

$$Var\left( N_{i} \right)=\sum_{k} Var\left( I\left( Z_{k}=i \right) \right)+\sum_{j\neq k} Cov\left( I\left( Z_{j}=i \right),I\left( Z_{k}=i \right) \right)\text{. }\left( S3 \right)$$

As shown previously in Eq. S1e, $P\left( Z_{k}=i \right)=p_{i}$, thus we have

$Var\left( I\left( Z_{k}=i \right) \right)=E\left( I^{2}\left( Z_{k}=i \right) \right)-\left[ E\left( I\left( Z_{k}=i \right) \right) \right]^{2}=p_{i}-p_{i}^{2}=p_{i}q_{i}.$ (S4)

Further,

$$Cov\left( I\left( Z_{j}=i \right),I\left( Z_{k}=i \right) \right)$$

$$=E\left( I\left( Z_{j}=i \right)I\left( Z_{k}=i \right) \right)-E\left( I\left( Z_{j}=i \right) \right)E\left( I\left( Z_{k}=i \right) \right),$$

where the calculation of $P\left( Z_{j}=i,Z_{k}=i \right)$ followed the standard Markov matrix property, after some tedious algebra, one can show that,

$P\left( Z_{k}=i,Z_{j}=i \right)=\pi^{\left| k-j \right|}p_{i}q_{i}$

Therefore, substituting the results in Eqs. S4 and S5 into Eq. S3 leads to

$$Var\left( N_{i} \right)=\sum_{k} Var\left( I\left( Z_{k}=i \right) \right)+\sum_{j\neq k} Cov\left( I\left( Z_{j}=i \right),I\left( Z_{k}=i \right) \right)$$

$$=mp_{i}q_{i}+2p_{i}q_{i}\sum_{j=1}^{m} \sum_{k=j+1}^{m} \pi^{k-j}, (S6)$$

in which, for a large *m*, we suggest an approximate and simple formula is

$$Var\left( N_{i} \right)\approx mp_{i}q_{i}+2mp_{i}q_{i}\frac{\pi}{1-\pi}$$

$$=mp_{i}q_{i}\frac{1+\pi}{1-\pi}$$

$=\delta mp_{i}q_{i}$, (S7)

where we define $\delta=\frac{1+\pi}{1-\pi}$ for simplicity.

*Derivation of Eq. 8 of the main text*

To show the result in Eq. 8 of the main text, using

$$Var\left( N_{i} \right)=E\left( N_{i}^{2} \right)-E\left( N_{i} \right)^{2}$$

$$=E\left( N_{i}^{2} \right)-\left( mp_{i} \right)^{2} (S8)$$

along with the result in Eq. S6 gives

$$E\left( N_{i}^{2} \right)=Var\left( N_{i} \right)+\left( mp_{i} \right)^{2}$$

$$\approx mp_{i}\left( \delta q_{i}+mp_{i} \right)$$

$$=mp_{i}\left( \delta\left( 1-p_{i} \right)+mp_{i} \right)$$

$$=mp_{i}\delta-mp_{i}^{2}\left( \delta-m \right)$$

$$=E\left( N_{i} \right)\delta-mp_{i}^{2}\left( \delta-m \right), (S9)$$

as a consequence, we have

$$p_{i}^{2}\approx\frac{E\left( N_{i}^{2} \right)-E\left( N_{i} \right)\delta}{m\left( m-\delta\right)}\text{,}$$

from which the desired result in Eq. 8 of the main text is evident and shown below

$$\Delta=1-\sum_{i=1}^{S} p_{i}^{2}\approx\frac{m^{2}-\sum_{i=1}^{S} E\left( N_{i}^{2} \right)}{m\left( m-\delta\right)} \left( S10 \right)$$

After removing the expectation operator, suppose we have found an estimator for the parameter $\pi$ or $\delta$, the proposed estimator for $\Delta$ becomes $1-\sum_{i} \frac{N_{i}\left( N_{i}-\hat{\delta} \right)}{m\left( m-\hat{\delta} \right)}$.

*Derivation of sampling covariance of the proposed model*

The covariance of abundance between two species *i* and *j* in the line-transect sampling under the Markov model is given by,

$$Cov\left( N_{i},N_{j} \right)=Cov\left( \sum_{l} I\left( Z_{l}=i \right),\sum_{k} I\left( Z_{k}=j \right) \right)$$

$$=\sum_{l=1}^{m} \sum_{k=1}^{m} Cov\left( I\left( Z_{l}=i \right),I\left( Z_{k}=j \right) \right)$$

$$=\sum_{l=1}^{m} \sum_{k=1}^{m} \left[ E\left( I\left( Z_{l}=i \right)I\left( Z_{k}=j \right) \right)-E\left( I\left( Z_{l}=i \right) \right)E\left( I\left( Z_{k}=j \right) \right) \right]. (S11)$$

.

Using the property of Markov transition probabilities and after some algebra manipulation, one can show that

$$E\left( I\left( Z_{l}=i \right),I\left( Z_{k}=j \right) \right)=\left\{ \begin{aligned} &0, &l=k; \\ &\left( 1-\pi^{\left| l-k \right|} \right)p_{i}p_{j}, &l\neq k, \end{aligned} \right. (S12)$$

for $i\neq j$, from which, for a large *m*, we have

$$Cov\left( N_{i},N_{j} \right)=Cov\left( \sum_{l} I\left( Z_{l}=i \right),\sum_{k} I\left( Z_{k}=j \right) \right)$$

$$=\sum_{l=1}^{m} \sum_{k=1}^{m} Cov\left( I\left( Z_{l}=i \right),I\left( Z_{k}=j \right) \right)$$

$$=-2\sum_{l=1}^{m} \sum_{k=l+1}^{m} \pi^{k-l}p_{i}p_{j}$$

$$\approx-mp_{i}p_{j}\frac{1+\pi}{1-\pi}$$

The last equality of Eq. S13 is the result of Eq. 4 in the main text, for which it is also one of the key formulas in our study.

*Derivation of the proposed new Rao’s quadratic diversity index*

Based on the above derivation in Eq. S8, we can see that $p_{i}p_{j}=-\frac{Cov\left( N_{i},N_{j} \right)}{\delta m}$, from which we can derive a nearly unbiased estimator of the Rao’s quadratic diversity index, under the Markov model context, by

$$Q\left( \boldsymbol{p} \right)=\sum_{i\neq j} d_{ij}p_{i}p_{j}$$

$$=-\sum_{i\neq j} d_{ij}\frac{Cov\left( N_{i},N_{j} \right)}{\delta m}$$

$$=-\sum_{i\neq j} d_{ij}\frac{E\left( N_{i}N_{j} \right)-E\left( N_{i} \right)E\left( N_{j} \right)}{\delta m}$$

$$=-\sum_{i\neq j} d_{ij}\frac{E\left( N_{i}N_{j} \right)-mp_{i}mp_{j}}{\delta m}$$

$$=-\frac{1}{m\delta}\sum_{i\neq j} d_{ij}E\left( N_{i}N_{j} \right)+\frac{m}{\delta}Q\left( \boldsymbol{p} \right). (S14)$$

Therefore, after removing the expectation operator, if we can find an estimator for $\delta$, we can derive the unbiased Rao’s quadratic diversity index under the non-independent sampling scenario as

$$\hat{Q}^{M}\left( \boldsymbol{p} \right)=\sum_{i\neq j} \frac{d_{ij}N_{i}N_{j}}{m\left( m-\hat{\delta} \right)}. \left( S15 \right)$$

The above equation is Eq. 10 in the main text. The associated Simpson index can be derived accordingly by assuming $d_{ij}=1$, which is actually the complement of Eq. 8 of the main text or the estimated $\Delta$ as showed above in Eq. S10. Specifically, to show this, when $d_{ij}=1$ in Eq. S15,

$$\hat{Q}^{M}\left( \boldsymbol{p} \right)=\sum_{i\neq j} \frac{N_{i}N_{j}}{m\left( m-\hat{\delta} \right)}$$

$$=\sum_{i} \frac{N_{i}}{m}\frac{m-N_{i}}{m-\hat{\delta}}$$

$$=\sum_{i} \frac{N_{i}}{m}\frac{m-N_{i}-\hat{\delta}+\hat{\delta}}{m-\hat{\delta}}$$

$$=\sum_{i} \frac{N_{i}}{m}\left( 1-\frac{N_{i}-\hat{\delta}}{m-\hat{\delta}} \right)$$

$$=1-\sum_{i} \frac{N_{i}}{m}\left( \frac{N_{i}-\hat{\delta}}{m-\hat{\delta}} \right). (S16)$$

*Derivation of Eq. 9 of the main text*

Using some key formulas (Eqs. S1a-S1e) introduced in the first place, we can also easily prove Eq. 7 in the main text as follows. Taking expectation of Eq. 6 gives

$$E\left( v \right)=E\left( \frac{1}{m-1}\sum_{k=2}^{m} \sum_{i=1}^{S} I\left( Z_{k}=i,Z_{k-1}=i \right) \right)$$

$$=\frac{1}{m-1}\sum_{k=2}^{m} \sum_{i=1}^{S} P\left( Z_{k}=i,Z_{k-1}=i \right)$$

$$=\frac{1}{m-1}\sum_{k=2}^{m} \sum_{i=1}^{S} P\left( \left. Z_{k}=i \right|Z_{k-1}=i \right)P\left( Z_{k-1}=i \right)$$

$$=\frac{1}{m-1}\sum_{k=2}^{m} \sum_{i=1}^{S} \left( \left( 1-\pi\right)p_{i}+\pi\right)p_{i}$$

$$=\left( 1-\pi\right)\sum_{i} p_{i}^{2}+\pi. (S17)$$

Therefore, Solow (2000)’s estimator *v* tended to overestimate the true $\pi$ value, because of the confounding influence of $\sum_{i=1}^{S} p_{i}^{2}$. However, as remarked in the main text, Solow (2000)’s estimator $v$ is still useful for deriving the proposed unbiased estimator of the true $\pi$ and valid for some cases (*e.g*., $\sum_{i=1}^{S} p_{i}^{2}$ is small).

To give the derivation of the proposed estimator of $\pi$, we merge two estimating equations (Eq. 7 and 8 in the main text) into a single one as

$$\frac{m^{2}-\sum_{i=1}^{S} N_{i}^{2}}{m(1-v)}=\frac{1}{1-\hat{\pi}}\left( m-\frac{1+\hat{\pi}}{1-\hat{\pi}} \right) (S18)$$

that only gets the estimator $\hat{\pi}$ involved. As a result, we can solve it as

$$\hat{\pi}=1-\frac{m\left( m+1 \right)\left( 1-v \right)+\sqrt{\left( m+1 \right)^{2}m^{2}\left( 1-v \right)^{2}-8m^{3}\left( 1-v \right)\hat{\Delta}}}{2m^{2}\hat{\Delta}}, (S19)$$

where $\hat{\Delta}=1-\sum_{i=1}^{S} \left( \frac{N_{i}}{m} \right)^{2}$. This is Eq. 9 in the main text.

**Unification of Rao's quadratic diversity index and Nei's genetic diversity**

In molecular ecology and genetics, Nei's nucleotide diversity and haplotype diversity indices are two of the most widely applied indices (Nei 1973, 1987, Nei and Li 1979, Nei and Kumar 2000). Actually they are termed as Rao’s quadratic diversity index and unbiased Simpson index, respectively, as proved below (Simpson 1949, Rao 1982, Nayak 1986, Botta-Dukat 2005, Chen et al. 2018).

The power of these two indices relies on the fact that they are unbiased estimators (Pons and Petit 1996, Hardy and Senterre 2007, Meirmans and Hedrick 2011), which is particularly true under large sample sizes. However, despite of its robust statistical property and wide application, a hidden issue is that organism sampling for genetic, molecular and ecological studies is that individuals are assumed to be independent. Both nucleotide and haplotype diversity indices are also established on the basis of this independent sampling assumption. This implies that, when field sampling is conducted in non-random ways, the conventional calculation of nucleotide diversity and haplotype diversity indices might become misleading or at least inaccurate.

In the calculation of traditional haplotype and nucleotide diversity indices, suppose among the *H* different haplotypes, their observed abundances in the sampled line transect with *m* individuals is given as $\boldsymbol{N}=\left\{ N_{1},N_{2},\ldots,N_{H} \right\}$, again we have $\sum N_{i}=m$. Then, the observed relative abundance of a specific haplotype *i* can be calculated as ${}^{0}{\hat{p}_{i}=}N_{i}/m$, forming a vector as $\hat{\boldsymbol{p}}\boldsymbol{=\{}{}^{0}{\hat{p}_{1},}{}^{0}{\hat{p}_{2},}\boldsymbol{\ldots,}{}^{0}{\hat{p}_{H}}\boldsymbol{\}}$. Then, the haplotype diversity index, under the context of random sampling of organisms, can be calculated as (Nei and Tajima 1981),

$$h\left( \hat{\boldsymbol{p}} \right)_{U}=\frac{m}{m-1}\left( 1-\sum_{i} \hat{p}_{\boldsymbol{i}}^{\boldsymbol{2}} \right)=\frac{m}{m-1}\left( 1-\sum_{i} \left( \frac{N_{i}}{m} \right)^{2} \right)=\frac{m}{m-1}\left( \sum_{i} \frac{N_{i}}{m}-\sum_{i} \left( \frac{N_{i}}{m} \right)^{2} \right)$$

$$=\frac{m}{m-1}\sum_{i} \frac{N_{i}}{m}\frac{m-N_{i}}{m}=\sum_{i} \frac{N_{i}}{m}\frac{m-N_{i}}{m-1}=\sum_{i} \frac{N_{i}}{m}\left( 1-\frac{N_{i}-1}{m-1} \right)=\sum_{i\neq j} \frac{N_{i}}{m}\frac{N_{j}}{m-1}$$

$$=1-\sum_{i} \frac{N_{i}}{m}\frac{N_{i}-1}{m-1}=\hat{\Delta}_{U}. (S20)$$

Suppose the observed site difference between a pair of haplotypes (*i* and *j*) is given by $\pi_{ij}$, then the traditional nucleotide diversity index, under the context of random sampling of organisms, is calculated as (Nei and Li 1979, Nei 1987),

$$\pi\left( \hat{\boldsymbol{p}} \right)=\sum_{i=2}^{H} \sum_{j=1}^{i-1} \pi_{ij}\hat{p}_{i}\hat{p}_{j}=\sum_{i=2}^{H} \sum_{j=1}^{i-1} \pi_{ij}\frac{N_{i}}{m}\frac{N_{j}}{m}. (S21)$$

This formula is identical to Rao's quadratic diversity metric as shown in Eq. 5 of the main text by setting the distance metrics $\pi_{ij}=d_{ij}$, $S=H$. By plugging observed relative abundance values into the formulae. In such a case, $\pi\left( \hat{\boldsymbol{p}} \right)=Q(\hat{\boldsymbol{p}})$.

References

Botta-Dukat, Z. 2005. Rao’s quadratic entropy as a measure of functional diversity based on multiple traits. Journal of Vegetation Science 16:533–540.

Chen, Y., Y. Wu, and T. Shen. 2018. Evaluation of the estimate bias magnitude of the Rao’s quadratic diversity index. PeerJ:e5211.

Hardy, O., and B. Senterre. 2007. Characterizing the phylogenetic structure of communities by an additive partitioning of phylogenetic diversity. Journal of Ecology 95:493–506.

Meirmans, P., and P. Hedrick. 2011. Assessing population structure: FST and related measures. Molecular Ecology Resources 11:5–18.

Nayak, T. 1986. An analysis of diversity using Rao’s quadratic entropy. Sankhya Series B 48:315–330.

Nei, M. 1973. Analysis of gene diversity in subdivided populations. PNAS 70:3321–3323.

Nei, M. 1987. Molecular Evolutionary Genetics. Columbia University Press, New York.

Nei, M., and S. Kumar. 2000. Molecualr evolutoin and phylogenetics. Oxford University Press, Oxford.

Nei, M., and W. Li. 1979. Mathematical model for studying genetic variation in terms of restriction endonucleases. PNAS 76:5269–5273.

Nei, M., and F. Tajima. 1981. DNA polymorphism detectable by restriction endonucleases. Genetics 97:145–163.

Pons, O., and R. Petit. 1996. Measuring and testing genetic differentiation with ordered versus unordered alleles. Genetics 144:1237–1245.

Rao, C. 1982. Diversity and dissimilarity coefficients: a unified approach. Theoretical Population Biology 21:24–43.

Simpson, E. 1949. Measurement of diversity. Nature 163:688.

**Additional Tables and Figures**

Table S1. A comparison on the fitted Markov non-independent parameter using Solow’s original estimator and the proposed one in the semi-numerical test in which the biomass information of 26 plant species in ultramafic soils of central Italy was used.


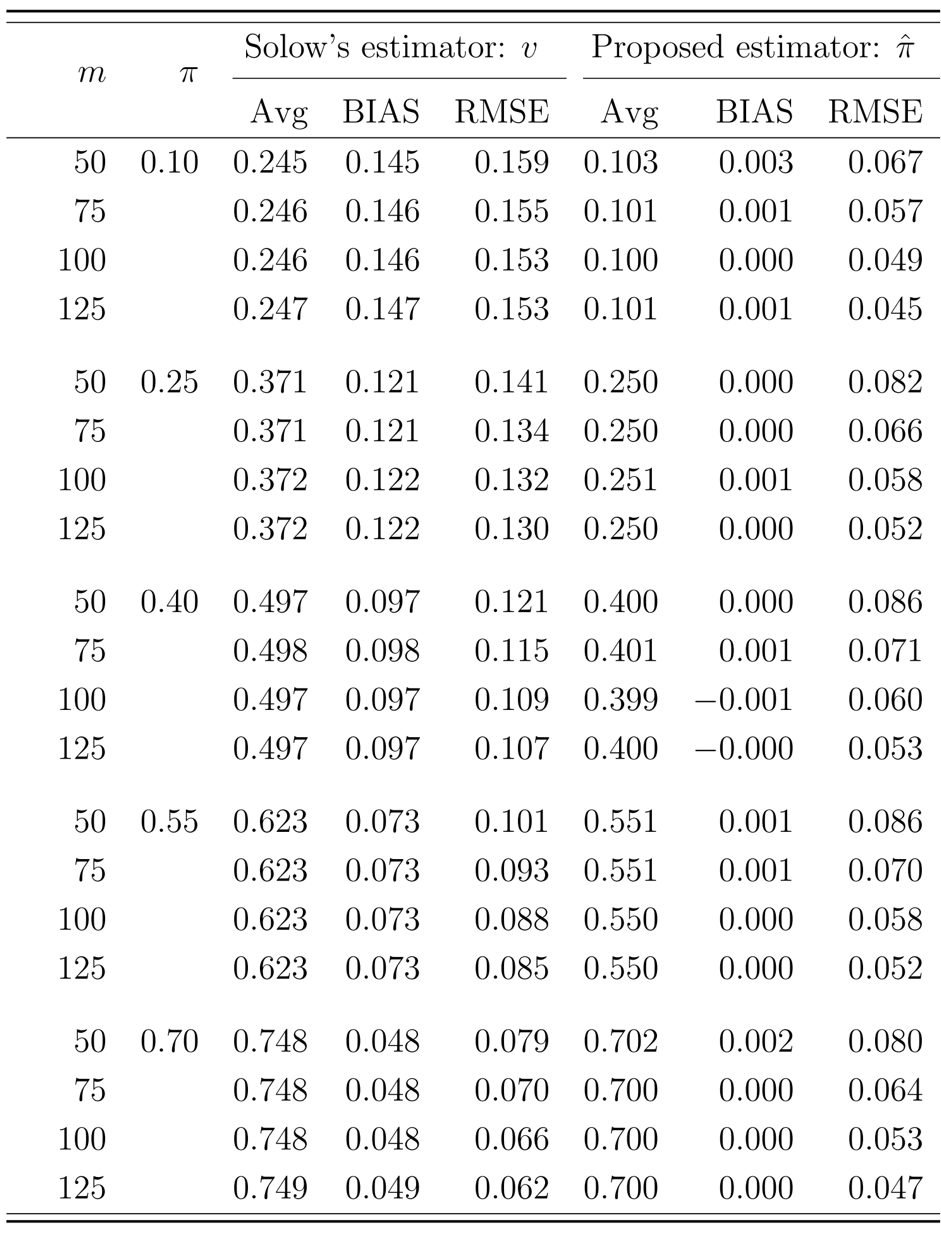


Table S2. Performance comparison between the proposed Rao’s estimator (Eq. 10) accounting for non-independent sampling of subsequent individuals, the original and unbiased Rao’s indices that only assume totally independent sampling of individuals if the sampling of individuals from the studied ecological community (i.e., 26 plant biomasses in ultramafic soils of central Italy) is assumed to be sequentially non-independent.


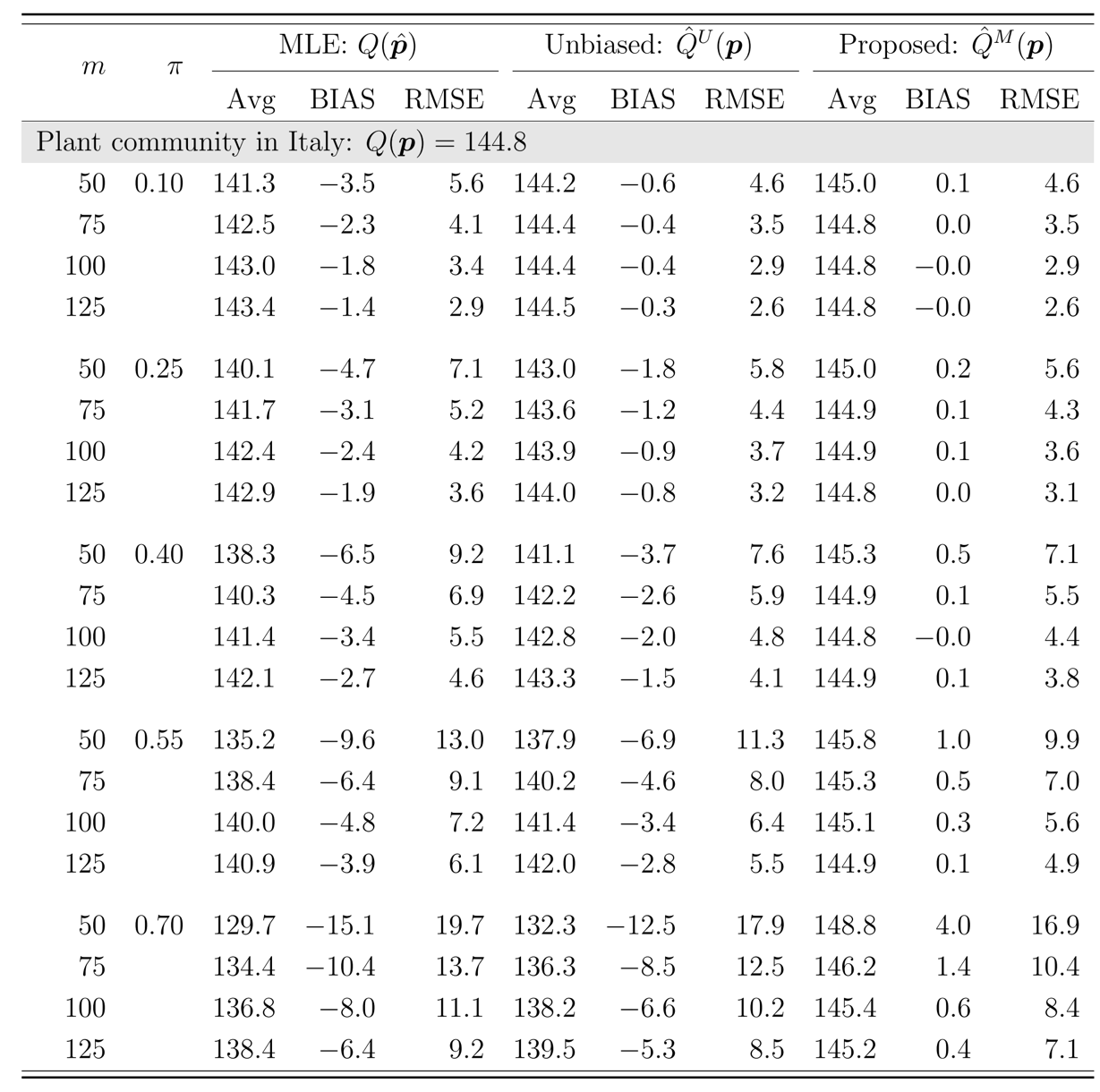


Table S3. Performance comparison between the proposed Gini-Simpson estimator (Eq. 8) accounting for non-independent sampling of subsequent individuals, the original and unbiased Gini-Simpson indices that only assume totally independent sampling of individuals if the sampling of individuals from the studied ecological community (i.e., 26 plant biomasses in ultramafic soils of central Italy) is assumed to be sequentially non-independent.


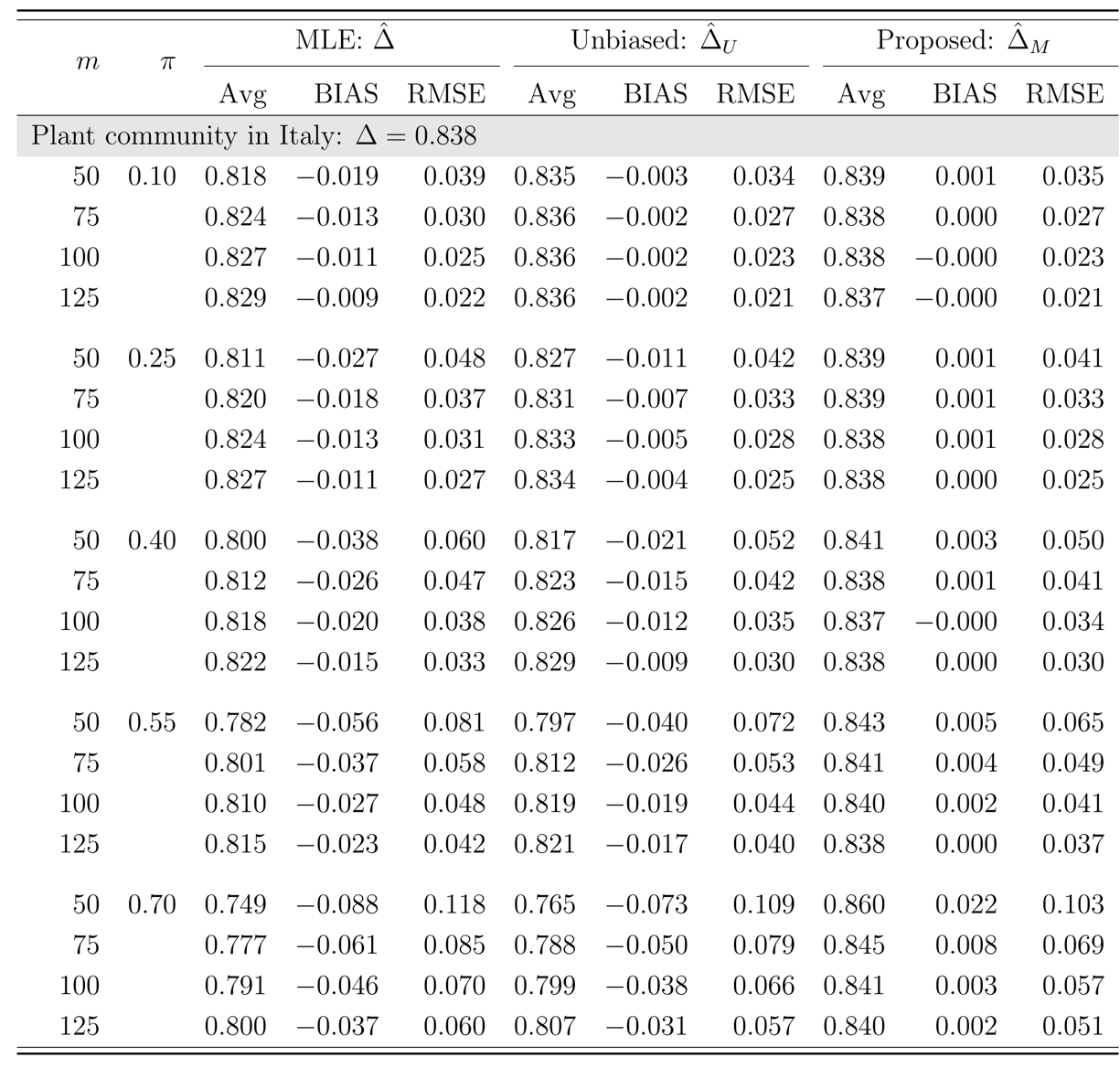


Table S4. Performance comparison between the proposed Gini-Simpson estimator (Eq. 8) accounting for non-independent sampling of subsequent individuals, the original and unbiased Gini-Simpson indices that are only valid for totally independent sampling of individuals if the sampling of individuals from the studied ecological community (i.e., 34 bat Phyllostomid genus abundances in Selva lacandona habitats) is assumed to be sequentially non-independent.


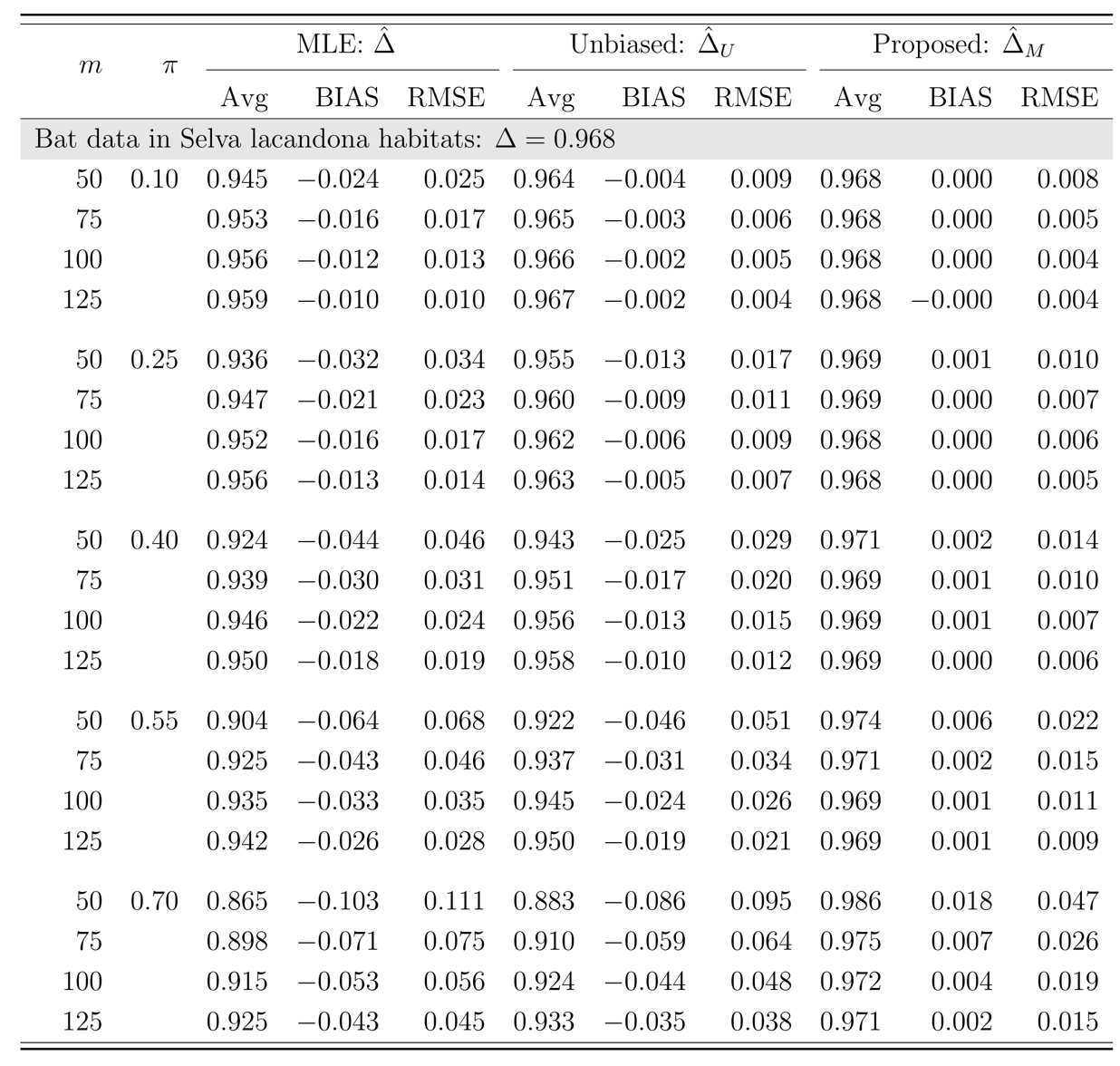


Table S5. Estimate average, averaged bias (BIAS), and RMSE of the ML (maximum likelihood), unbiased Rao’s index, and the proposed estimators in the tree data sampled from the line transects in the 50-ha BCI forest plot.


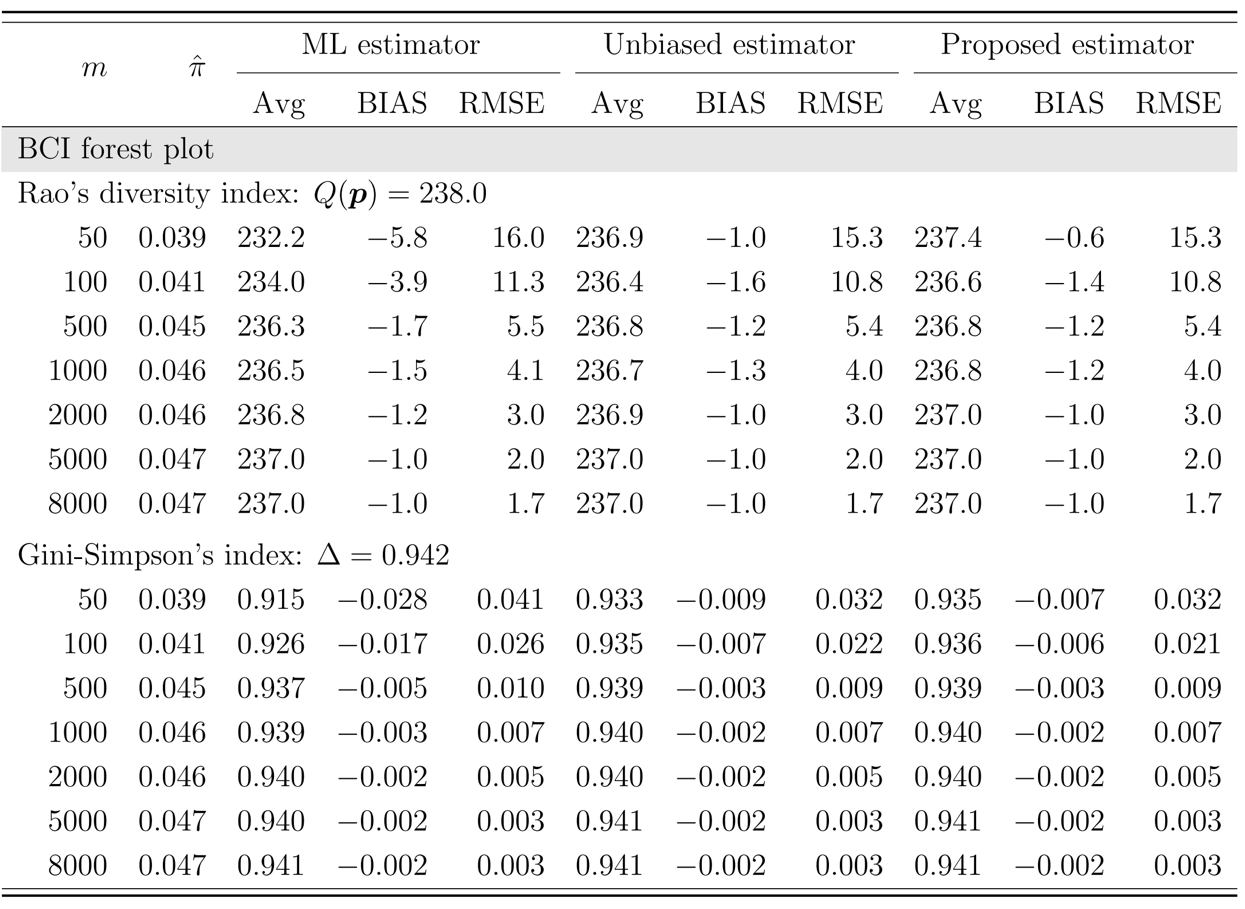


Table S6. Estimate average, averaged bias (BIAS), and RMSE of the ML (maximum likelihood), unbiased Rao’s index, and the proposed estimators in the tree data sampled from the line transects in the 50-ha HSD forest plot.


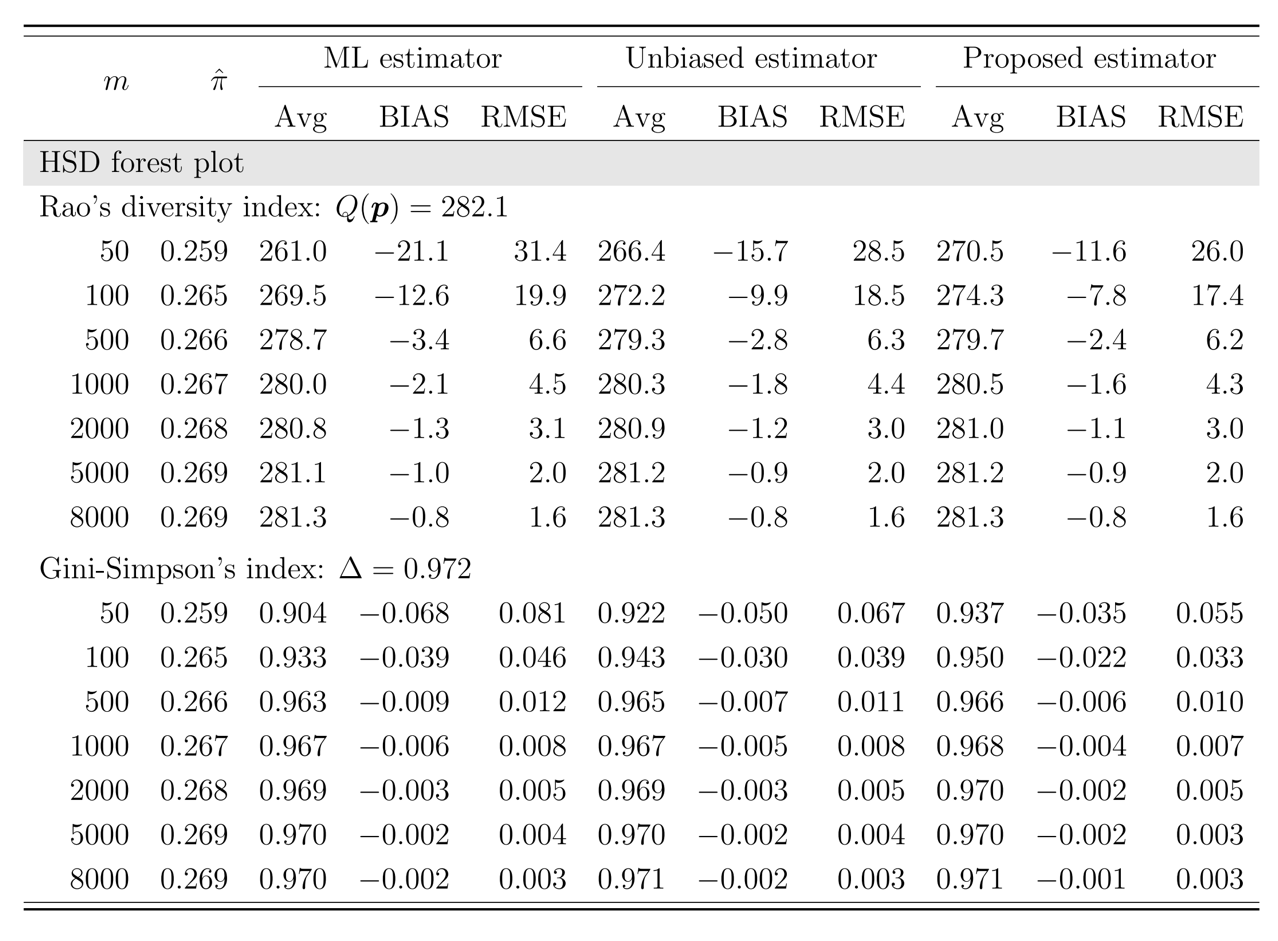


Table S7. Estimate average, averaged bias (BIAS), and RMSE of the ML (maximum likelihood), unbiased Rao’s index, and the proposed estimators from the line transects in the territory of Australia for Acacia species.


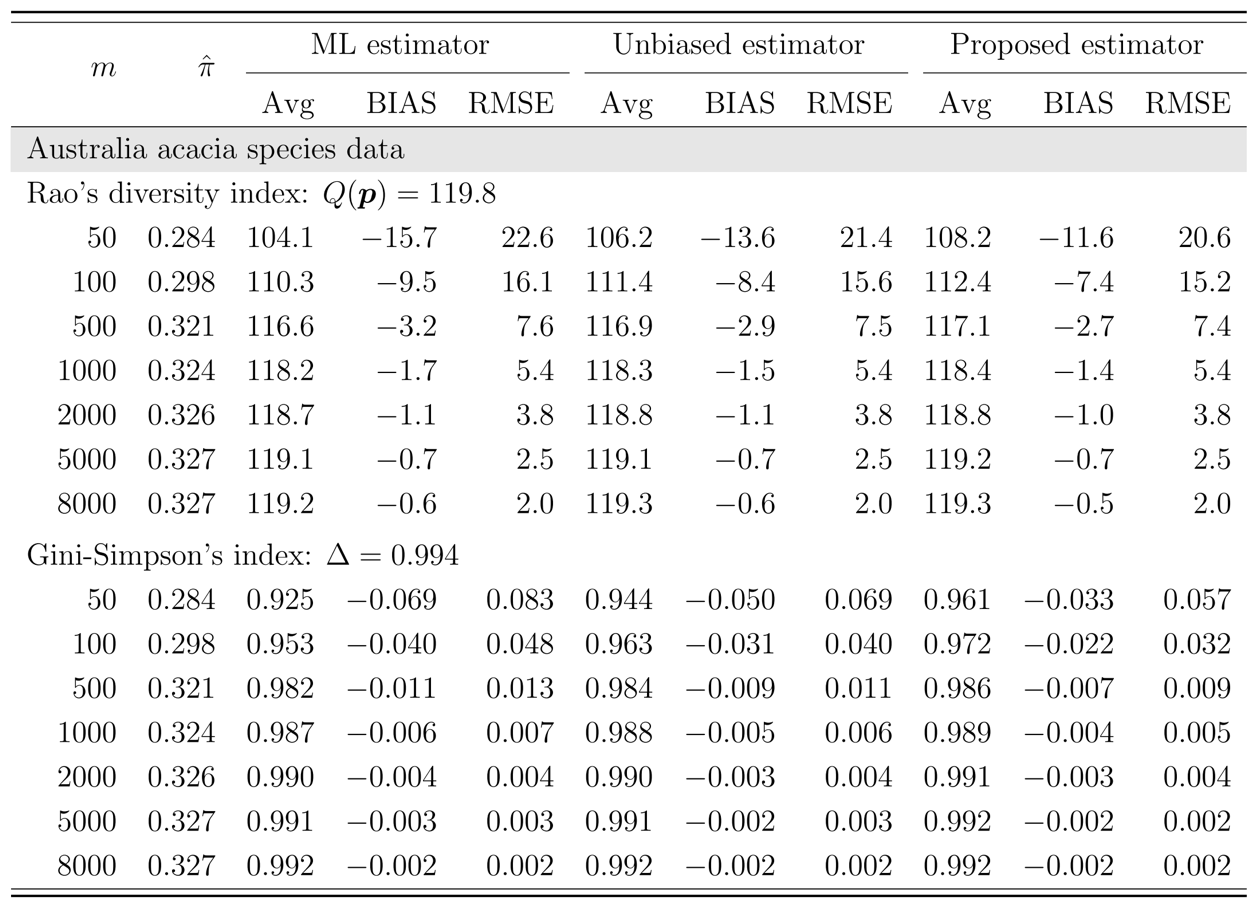

Supplement: Supplementary file 1 [file DataSheet_1.docx]
